# Methanolic Extract and Brominated Compound from the Brazilian Marine Sponge *Aplysina fulva* Are Neuroprotective and Modulate Inflammatory Profile of Microglia

**DOI:** 10.3390/md22060235

**Published:** 2024-05-22

**Authors:** Catarina de Jesus Nunes, Cinthia Cristina Santos, Erica Novaes Soares, Irlã Santos Lima, Uesley Vieira Alves, Emílio Lanna, Ronan Batista, Ravena Pereira do Nascimento, Silvia Lima Costa

**Affiliations:** 1 Laboratory of Neurochemistry and Cell Biology (LabNq), Department of Biochemistry and Biophysics, Institute of Science and Health, Federal University of Bahia, Salvador 40231-300, Bahia, Brazil; catarinanunes@ufba.br (C.d.J.N.); cinthia.santos@ufba.br (C.C.S.); ericasoares@ufba.br (E.N.S.); irlalima@ufba.br (I.S.L.); ravenanascimento@ufba.br (R.P.d.N.); 2 Laboratory of Research in Bioactive Substances (LAPESBI), Department of Organic Chemistry, Institute of Chemistry, Federal University of Bahia, Salvador 40170-115, Bahia, Brazil; uesleyvieira@outlook.com (U.V.A.); ronbatis@ufba.br (R.B.); 3 Biology Institute, Federal University of Bahia, Salvador 40170-115, Bahia, Brazil; emiliolanna@gmail.com; 4 National Institute of Translational Neuroscience, Rio de Janeiro 21941-902, Rio de Janeiro, Brazil

**Keywords:** marine sponges, neuroprotection, anti-neuroinflammatory, *Aplysina fulva*, 3,5-dibromoverongiaquinol dimethyl ketal

## Abstract

Neurodegenerative diseases involve neuroinflammation and a loss of neurons, leading to disability and death. Hence, the research into new therapies has been focused on the modulation of the inflammatory response mainly by microglia/macrophages. The extracts and metabolites of marine sponges have been presented as anti-inflammatory. This study evaluated the toxicity of an extract and purified compound from the Brazilian marine sponge *Aplysina fulva* as well as its neuroprotection against inflammatory damage associated with the modulation of microglia response. PC12 neuronal cells and neonatal rat microglia were treated with the methanolic extract of *A. fulva* (AF-MeOH, 0.1–200 μg/mL) or with its purified dimethyl ketal of 3,5-dibromoverongiaquinol (AF-H1, 0.1–100 μM). Cytotoxicity was determined by MTT tetrazolium, Trypan blue, and propidium iodide; microglia were also treated with the conditioned medium (CM) from PC12 cells in different conditions. The microglia phenotype was determined by the expression of Iba-1 and CD68. AF-MeOH and AF-H1 were not toxic to PC12 or the microglia. Inflammatory damage with *Escherichia coli* lipopolysaccharide (LPS, 5 μg/mL) was not observed in the PC12 cells treated with AF-MeOH (1–10 μg/mL) or AF-H1 (1–10 μM). Microglia subjected to the CM from PC12 cells treated with LPS and AF-MeOH or AF-H1 showed the control phenotype-like (multipolar, low-CD68), highlighting the anti-neuroinflammatory and neuroprotective effect of components of this marine sponge.

## 1. Introduction

In the central nervous system (CNS), glial cells have a fundamental role in controlling homeostasis [1]. The breakdown of homeostasis implies an immediate response and change in the cellular phenotype, going from quiescent to reactive, which generates the inflammatory response, also called neuroinflammation [2]. After inflammatory and other damage, glial cells, especially microglia, react and release inflammatory mediators that generate communication between themselves and other CNS cells. In turn, if the microglial response is not well-controlled, it may contribute to the pathogenesis of neuroinflammation observed in several CNS pathologies, mainly in neurodegenerative diseases such as Alzheimer’s disease, Parkinson’s disease, and multiple sclerosis [3,4]. Although neuroinflammation is a protective tool, this event, when prolonged, tends to generate neurotoxicity and demyelination, favoring the emergence and progression of neurodegenerative diseases [2] The change in microglia phenotype is typically characterized as pro-inflammatory (M1) or anti-inflammatory (M2). In the M1 profile, the production of cytokines and chemokines such as interleukin 6 (IL-6), IL-1β, tumor necrosis factor alpha (TNF-α), and chemokine L2 (CCL2) occurs, which favor the maintenance of a neurotoxic environment, generating neuroinflammation and neuronal death. Thus, the research and development of neuroprotective medicines, which have mechanisms of action based on neuroprotective and anti-inflammatory activities, constitute the most important strategies in the development of new therapies for neurodegenerative processes [5].

In this context, natural products have served as the main therapeutic approach for most human diseases and are essential for the development of new medicines. In fact, more than 60% of recently created medicines fall into the categories of natural products, derived from, or inspired by natural products [6]. The marine environment offers a rich source of structurally unique natural compounds with strong bioactivities, many of which belong to completely new chemical classes that are not present in terrestrial sources [7].

Sea sponges are the richest source of potential marine pharmaceuticals among marine animals, representing almost 30% of all natural marine products found [8]. At the same time, they have also attracted attention as a promising source for the development of drugs targeting neuroinflammation [9,10]. In our continuous search for new neuroprotective compounds of natural origin, we selected the sponge *Aplysina fulva* (Porifera, Demospongiae), easily found on the coast of Salvador, Bahia, Brazil, for studies focused on evaluating the neuroprotective properties of its methanolic extract and its main brominated alkaloid.

This study evaluated the toxicity of a methanolic extract of the Brazilian marine sponge *A. fulva* and its purified brominated compound for neuronal cells and neuroprotection against inflammatory damage as well as the impact on the inflammatory profile of microglia. The set of results highlight the anti-neuroinflammatory and neuroprotective effects of the components of this marine sponge, providing new studies on the mechanisms and combinations for therapeutic use.

## 2. Results

### 2.1. The Methanolic Extract of Aplysina fulva (AF-MeOH) and Its Dereplication

The marine sponge *A. fulva* was collected at Porto da Barra Beach in Salvador, Bahia, Brazil, the same locality as previously reported [11]. Once identified, the material was extracted with methanol and ethyl acetate (AcOEt), and the combined organic solution was evaporated and suspended in water for extraction with ethyl acetate. The AcOEt phase was then concentrated and dissolved in methanol, defatted with hexane, and evaporated to give the corresponding methanolic extract (AF-MeOH).

**Figure 1:**
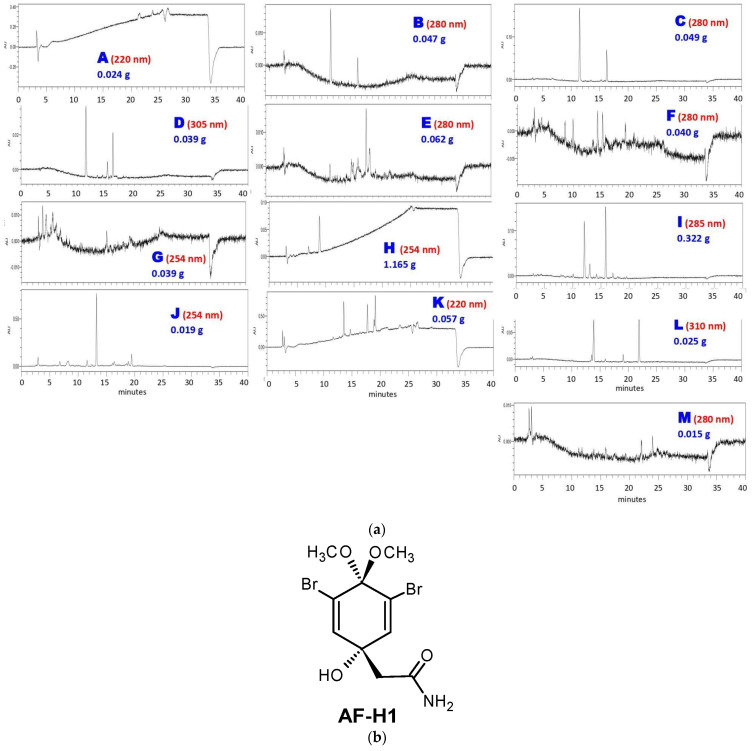
Sephadex^®^ column fractionation of AF-MeOH. (**a**) HPLC analyses of grouped fractions A–M under UV detection and their corresponding masses. (**b**) The structural formula of the major constituent 5-dibromoverongiaquinol dimethyl ketal (AF-H1) isolated from the grouped fraction H.

The next step was subjecting AF-MeOH to LH-20 Sephadex^®^ column fractionation. This approach afforded 330 fractions that were grouped into thirteen fractions A–M, taking into account their chromatogram profile similarity, UV absorption regions, and low-resolution masses (Figure 1a). As could be noted, the major content was found in fraction H (1.165 g), which was pure under ^1^H and ^13^C NMR analysis, and was identified as 3,5-dibromoverongiaquinol dimethyl ketal (AF-H1, Figure 1b).

Both the AF-MeOH and AF-H1 samples were assessed for their cytotoxicity to neuronal cells as well as for their neuroprotection effects against inflammatory damage associated with the modulation of microglia response.

### 2.2. A. fulva Methanolic Extract (AF-MeOH) and Its Brominated Compound 3,5-Dibromoverongiaquinol (AF-H1) Are Not Cytotoxic to PC12 Neuronal Cells

**Figure 2:**
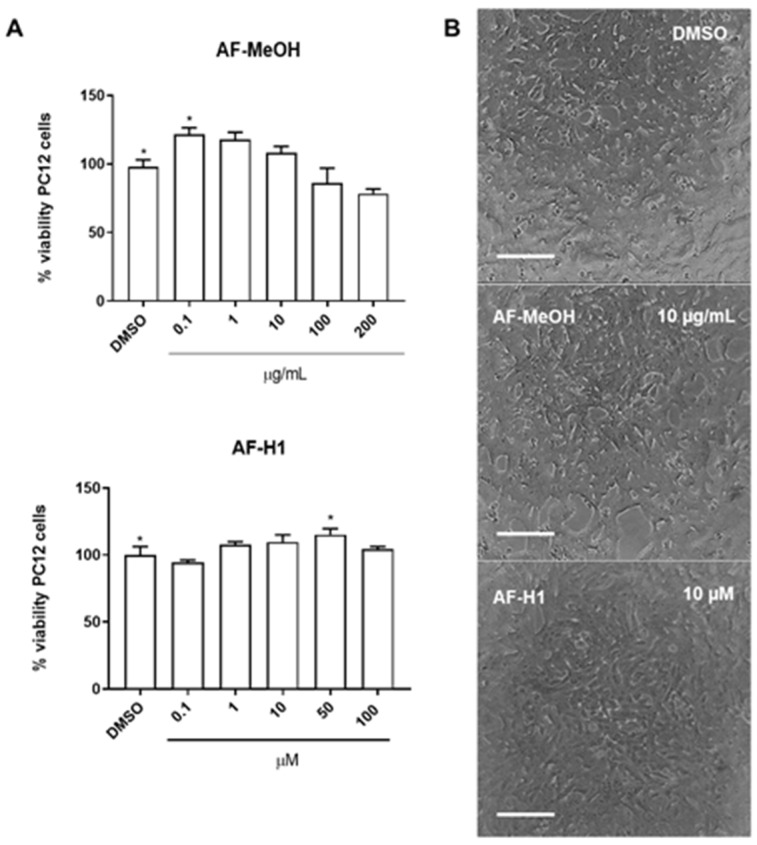
Cytotoxicity of the methanolic extract (**A**) from *A. fulva* (AF-MeOH) and its purified compound dimethyl ketal compound of 3,5-dibromoverongiaquinol (AF-H1) (**B**) to PC12 cell cultures. Cell viability was determined using the MTT assay 24 h after treatment with AH-F1 (0.1 to 200 μM); the results are expressed as a percentage of the control (DMSO 0.1%), considered 100% (* *p* < 0.05, ANOVA followed by the Kruskal–Wallis test). Representative images of the morphology of the PC12 cells in control conditions or after exposure to AF-MeOH (1 and 10 μg/mL) (**A**) or AF-H1 (1 and 10 μM) (**B**) by phase contrast microscopy; obj ×20; scale bar = 100 μm).

The cytotoxicity of AF-MeOH (0.1, 1, 10, 100 and 200 μg/mL) and its purified compound AF-H1 (0.1, 1, 10, 50, and 100 μM) was investigated in PC12 cells after 24 h of treatment by the 3-(4,5-dimethylthiazol-2-yl)-2,5-diphenyltetrazolium bromide (MTT) assay. Neither AF-MeOH nor compound AF-H1 (Figure 2B) showed toxicity to PC12 cells under these conditions. PC12 cells in the control cultures presented typical bipolar morphology. Cells treated with AF-MeOH or compound AF-H1 presented a preserved phenotype, without signs of toxicity.

### 2.3. Cytotoxicity of Methanolic Extract of A. fulva (AF-MeOH) and 3,5-Dibromoverongiaquinol Dimethyl Ketal (AF-H1) on Microglia

**Figure 3:**
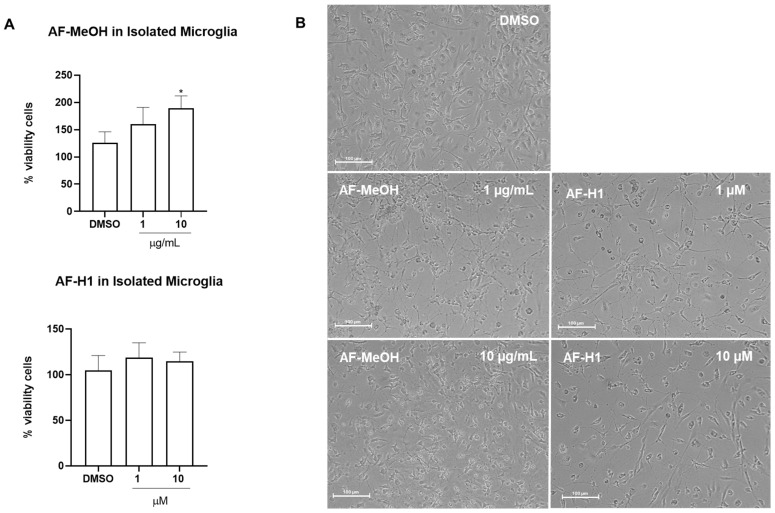
Cytotoxicity and morphology analysis of rat microglia subjected to treatment with the methanolic extract of *A. fulva* AF-MeOH and its dimethyl ketal compound 3,5-dibromoverongiaquinol (AF-H1). (**A**) Cell viability was determined using the MTT assay 24 h after treatment with AF-MeOH (0.1, 1, and 10 μg/mL) or AF-H1 (0.1, 1, and 10 μM); the results are expressed as a percentage of the control (DMSO 0.1%), considered 100% (* *p* < 0.05). (**B**) Morphology of microglia in control conditions (DMSO 0.1%) or after 24 h of exposure with treatments with AF-MeOH (1 and 10 μg/mL) or AF-H1 (1 and 10 μM) by phase contrast microscopy; Obj. ×20; bar scale 100 μm.

Microglia obtained from the cerebral cortex of rats were directly exposed to AF-MeOH (0.1–10 μg/mL) or AF-H1 (0.1–10 μM); cell viability was evaluated 24 h after treatment by the MTT test, and changes in phenotype were investigated by phase contrast microscopy (Figure 3B). Treatment with AF-H1 (0.1, 1, and 10 μM) did not induce changes to microglia viability compared to the control cultures (DMSO 0.1%); in contrast, a significant increase in the proportion of viable cells was observed in the microglia cultures treated with AF-MeOH at the highest concentration tested (10 μg/mL) (Figure 3A). It was also possible to observe morphological changes in the microglia exposed to AF-MeOH (10 μg/mL) (Figure 3B). In cultures under control conditions, the majority of microglia presented a morphology typical of the so-called quiescent state, with a contracted cell body and thin cellular processes; on the other hand, in cultures treated with AF-MeOH (10 μg/mL), there was an increase in cellularity and the majority of microglia presented a morphology consistent with an activated phenotype, with an amoeboid cell body and thicker processes.

### 2.4. Protective Effect of AF-MeOH and AF-H1 on PC-12 Neuronal Cells Subjected to Inflammatory Damage and Impact on Microglia Response

**Figure 4:**
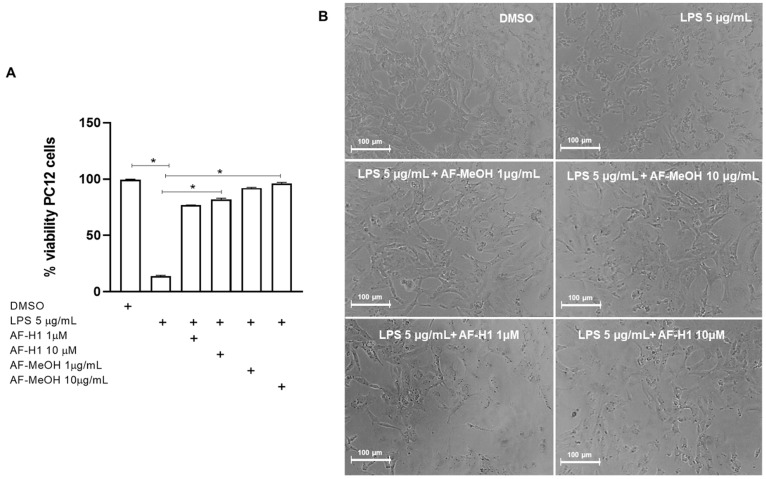
Analysis of neuroprotection induced by the methanolic extract of *A. fulva* AF-MeOH and its dimethyl ketal compound 3,5-dibromoverongiaquinol (AF-H1) purified in PC12 cell cultures against inflammatory damage. (**A**) Cell viability was determined after staining with Trypan blue in cultures 24 h after treatment with LPS (5 μg/mL) for 12 h and treated with LPS and subsequently treated with AF-MeOH (1 and 10 μg/mL) or AF-H1 (1 and 10 μM) for 24 h. The results are expressed as a percentage of the control (DMSO 0.1%), considered 100% (* *p* < 0.05, ANOVA followed by the Kruskal–Wallis test). Values were expressed as the means ± SD (*n* = 3). (**B**) Representative phase-contrast photomicrographs of the cultures in control, LPS-treated, and LPS-treated conditions with AF-MeOH (1 and 10 μg/mL) or AF-H1 (1 and 10 μM); Obj. ×20; scale bar = 100 μm.

To characterize the effect of AF-MeOH or AF-H1 treatments of neuronal cells exposed to inflammatory damage on the cell viability and inflammatory profile of microglia, cultures of the PC12 lineage were subjected to inflammatory damage by LPS (5 μg/mL) for 12 h, and then the cells were treated with AF-MeOH (1 and 10 μg/mL) or AF-H1 (1 and 10 μM) for 24 h. The Trypan blue dye exclusion assay (Figure 4) showed a significant (86%) reduction in the proportion of viable cells observed in PC12 cultures exposed to LPS (5 μg/mL) compared to the control cultures (0.1% DMSO). On the other hand, in the PC12 cultures exposed to damage and treated with AF-MeOH or AF-H1, at both concentrations tested, the proportion of viable cells was similar to that in cultures under control conditions and therefore significantly higher than the cultures exposed to LPS (Figure 4A). Treatment with AF-MeOH showed better protection for cells exposed to inflammatory damage compared to treatment with the substance AF-H1. In cell cultures under control conditions (0.1% DMSO), PC12 cells presented lineage-typical morphology with a polygonal cell body and two to three short cellular processes. In cultures exposed to LPS, there was a reduction in cellularity and the remaining adherent cells presented a contracted cell body typical of damage. On the other hand, in cultures exposed to LPS and treated with AF-MeOH or AF-H1, the morphology of PC12 cells was preserved (Figure 4B).

**Figure 5:**
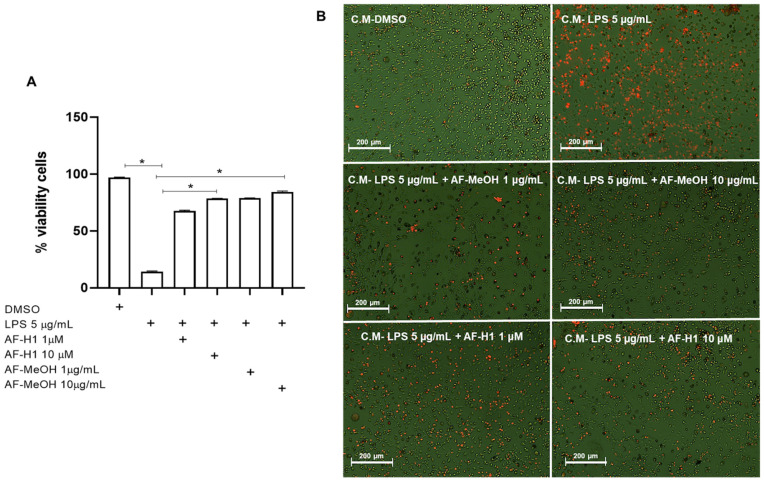
Analysis of the viability of rat microglia after exposure to conditioned medium (CM) from PC12 cells subjected to inflammatory damage and treated with a methanolic extract of *A. fulva* (AF-MeOH) and its dimethyl ketal compound 3,5-dibromoverongiaquinol (AF-H1). (**A**) Proportion of viable microglia was determined by staining with propidium iodide 24 h after treatment with the CM from PC12 cultures in control conditions (CM-DMSO), exposed to LPS (CM-LPS 5 μg/mL), exposed to LPS and treated with AF-MeOH (CM-LPS 5 μg/mL + AF-MeOH 10 μg/mL), or exposed to LPS and treated with AF-H1 (CM-LPS 5 μg/mL + AF-H1 10 μM).The results are expressed as a percentage of the control (CM-DMSO), considered 100% (* *p* < 0.05, ANOVA followed by the Kruskal–Wallis test). (**B**) Representative photomicrographs obtained by phase contrast associated with fluorescence microscopy of microglial cultures in the different conditions; Obj. ×20; scale bar = 200 μm; non-viable cells appear stained with the bright orange typical of the DNA intercalating exclusion dye propidium iodide.

**Figure 6:**
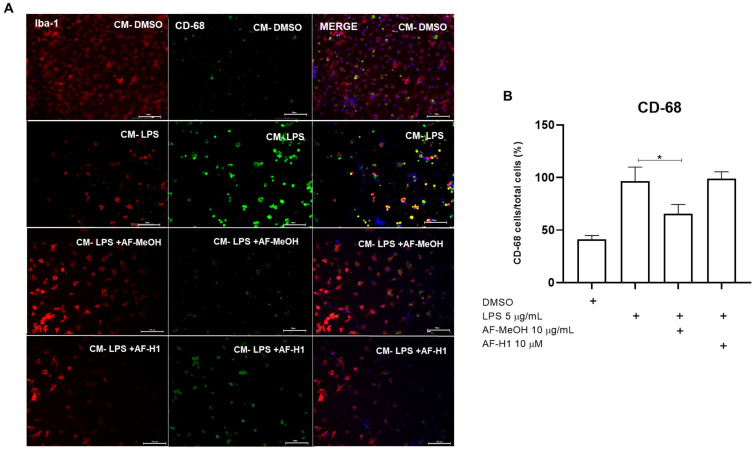
Immunocytochemistry for CD-68 and Iba-1 in microglia cultures subjected to treatment with PC12 conditioned medium (CM) after inflammatory damage. (**A**) Representative micrographs of microglia immunostained for the cytoskeletal protein Iba-1 and for the inflammatory maker CD-68 in cultures in control conditions or treated for 24 h with CM from PC12 cultures in the control conditions (CM-DMSO), exposed to LPS (CM-LPS 5 μg/mL), exposed to LPS and treated with AF-MeOH (CM-LPS 5 μg/mL + AF-MeOH 10 μg/mL), or exposed to LPS and treated with AF-H1 (CM-LPS 5 μg/mL + AF-H1 10 μM)); Obj. ×20; scale bar = 100 μm. (**B**) Quantification of microglia (Iba-1^+^) in the cultures and CD-68 positive cells (CD68^+^/Iba-1^+^) in each condition; statistical analysis carried out using the GraphPad Prism software (version 8.0, Boston, MA, USA) and the results expressed as a percentage of the control (DMSO 0.1%), considered 100% (* *p* < 0.05, ANOVA followed by the Kruskal-Wallis test).

To verify the effect of treatments with AF-MeOH (1 and 10 μg/mL) or AF-H1 (1 and 10 μM) on PC12 neuronal cells subjected to inflammatory damage on the microglial activation state, an indirect interaction assay between these cell populations via conditioned medium (CM) was performed. For this, the rat cortical microglia were maintained in control conditions (DMSO) or subjected to treatment for 24 h to CM from PC12 cell cultures exposed only to LPS, or treated with AF-MeOH (1 and 10 μg/mL), or AF-H1 (1 and 10 μM), or both, and the cell viability and morphology were evaluated after 24 h (Figure 5 and Figure 6). There was a significant increase in the PI-stained microglia cells treated with the CM from PC12 cell cultures treated with LPS compared to the control cultures (DMSO) (Figure 5A,B). Cultures exposed to damage and treated with AF-MeOH or AF-H1 at concentrations of 1 and 10 μg/mL or 1 and 10 μM showed a proportion of viable cells similar to the control cultures, being considerably higher than cultures that were subjected only to LPS.

To characterize microglia activation and phenotype, the microglia were maintained in control conditions (DMSO) or subjected to treatment for 24 h to CM from the PC12 cell cultures exposed only to LPS, or to LPS and treated with AF-MeOH (10 μg/mL) or AF-H1 (10 μM). Subsequently, immunocytochemistry assays using antibodies for the cytoskeletal protein Iba-1 and for the inflammatory maker CD68 were performed (Figure 6). It was observed that the majority of microglia cells in cultures under the control conditions exhibited typical quiescent morphology, and few cells expressing the proinflammatory marker CD68. In cultures exposed to CM from PC12 cells treated with LPS (CM-LPS), there was a reduction in the number of adherent viable microglia compared to the control cultures, with the majority (64%) of cells expressing CD68, typical characteristics of an activated phenotype after damage. Moreover, there was a reduction in CD68 expression in the microglia exposed to the CM of PC12 cells treated with LPS in combination with AF-MeOH (CM-LPS + AF-MeOH) compared to the control cultures exposed to CM from PC12 cells subjected to inflammatory damage with LPS. 

## 3. Discussion

In this study, we used PC12 cells to test the neurotoxicity and protection of AF-MeOH and its brominated compound against inflammatory damage, a neuronal cell lineage commonly used to study neurobiological activities as they have characteristics of mature neurons, which allows for the evaluation of neuronal interactions and responses [12]. On the other hand, microglia obtained from primary cultures of the cerebral cortex of neonatal rats were adopted to investigate the impact of these *A. fulva* constituents on the response of these cells by considering the change in phenotype and the expression of pro-inflammatory markers associated with CNS pathology damage [13].

To induce neuroinflammatory damage, LPS was used in this study. Studies in vitro and in vivo have shown that inflammatory damage induced by LPS activates macrophages that induce inflammatory storms as well as oxidative stress [14]. Furthermore, LPS acts on TLR4 receptors, producing inflammatory molecules such as NF-α, IL-6, and IL-1β, an increased activity of iNOS, COX-2, β-secretase, γ-secretase, Aβ accumulation as well as oxidative stress [15], therefore, it is a good model for inducing neuroinflammation and allowing for the study of specific pathways associated with this phenomenon.

It was observed that AF-MeOH did not present toxicity to the PC12 cells after 72 h of exposure. As described in Ibrahim et al. (2010) [16], methanolic extracts from marine sponges show less toxicity and increased viability to cells as methanol is a polar solvent, and due to its affinity, there is a greater disposition of compounds such as phenols and alkaloids, in addition to containing a wide variety of secondary metabolites, which is consistent with our findings.

Since marine sponge extracts contain several compounds, the *Aplysina* genus has been widely investigated due to the biological activities of its secondary metabolites [17]. According to Lira and collaborators (2011) [18], *Aplysina* species have a moderate amount of sterols and a low level of terpenes; however, a significant series of brominated derived from tyrosine were observed. These metabolites originate through the process of enzymatic brominization, which consists of the halogenation mechanism present in certain marine organisms such as sponges as a method of defense against predators. In terms of the main biological activities, their antimicrobial and anti-inflammatory potential stand out.

AF-H1 was isolated from the MeOH extract of the species *A. fulva* (AF-MeOH), a chemical compound belonging to the class of brominated alkaloids, commonly produced by marine organisms such as sponges. As the name suggests, brominated alkaloids have bromine atoms in their molecular structure and are commonly found in marine sponges of the order Verongida and genus Aplysina, resulting from the enzymatic diversion of aerophobin-2 and iso-fistulin-1 after a harmful stimulus in the environment [19]. As described by Thoms et al. (2006) [20], AF-H1 has biological properties such as antibacterial, antitumor, and anti-inflammatory activity. In our study, PC12 cells were exposed to increasing concentrations from 0.1 to 100 μM for 24 h, showing no toxicity or induction of changes in cell morphology. The toxicity of AF-MeOH and AF-H1 was also tested on the microglia, CNS immune effector cells, without observing cytotoxicity or morphological changes.

Cells of the PC12 lineage were exposed to LPS (5 μg/mL) for 12 h and then treated with the MeOH extract of *A. fulva* and its compound AF-H1 at subtoxic concentrations. It was observed that after exposure to damage, the cells showed morphological changes such as vacuolization and retraction of their cell body, in addition to a reduction in viability already described in Wiatrak and collaborators (2020) [12]. In contrast, PC12 cells subjected to inflammatory damage with LPS and subsequently treated with AF-MeOH and AF-H1 showed preserved morphology and remained mostly viable. According to Alghazwi et al. (2020) [9], marine organisms present neuroprotective compounds, where the junction and higher concentration of substances that demonstrate these activities are present in the crude extracts as well as the interaction between them. We therefore propose that the best protection results in PC12 cells subjected to damage and treated with AF-MeOH were influenced by the interaction of the compounds present in the crude extract when compared to treatment with the isolated compound AF-H1. Low-resolution mass spectra of many peaks observed in the chromatograms of grouped fractions A–M (data not published) suggest the presence of other dibrominated compounds as minor constituents of AF-MeOH, in agreement with previously reported data [21]. Taking into account that brominated natural products from marine sources have already displayed neuroprotective properties [9,10], one can infer that the minor brominated compounds of AF-MeOH might act as enhancer agents for the neuroprotective action of AF-H1, thus justifying the better performance of AF-MeOH in comparison with AF-H1. Jimenez-Romero and collaborators (2014) [22] stated that the neuroprotective profile of extracts and compounds isolated from the *Aplysina* genus may be related to their anti-inflammatory properties, helping to reduce inflammation and consequently limiting cellular damage, corroborating our findings.

Once we observed the protective effect of AF-MeOH and AF-H1 on PC12 cells subjected to inflammatory damage with LPS, we began to evaluate, through indirect interaction, the impact on the microglial activation phenotype in Wistar rats using PC12 conditioned medium. A cytotoxicity test of these substances was carried out on rat microglia. As in the PC12 cells, there was no toxicity or morphological changes; however, the concentration of 10 μg/mL of the MeOH extract of *A. fulva* showed an increase in cell viability in cultures of microglia isolated from rats, which could be interpreted as inducing proliferation.

In agreement with our results, Catanesi et al. (2021) [23] demonstrated the low toxicity of marine compounds on microglia. Microglia exposed to conditioned medium from PC12 cells (MC-PC12) subjected to inflammatory damage with LPS showed reduced viability as well as changes in morphology, assuming amoeboid characteristics indicating activation toward a characteristic pro-inflammatory profile. However, microglia exposed to MC-PC12 subjected to inflammatory damage and treated with AF-MeOH or AF-H1 showed an increase in viability and acquired a more branched phenotype typical of regulatory function. As described by Yu et al. (2021) [24], a medium conditioned with substances that have anti-inflammatory activity tends to contain neurotrophic and antiapoptotic factors that can regulate immune responses and protect cells against injury, which validates our findings. Given that neurotrophic factors play important roles in neuroprotection, we understand that both the MeOH extract of *A. fulva* and its isolated compound AF-H1 were able to modulate the release of neurotrophic factors in the PC12 conditioned medium adopted, and consequently changed the microglial profile from pro-inflammatory to anti-inflammatory.

Together, these results demonstrate that the MeOH extract from the marine sponge *A. fulva* and its purified compound (AF-H1), in addition to the neuroprotective effect, could influence the microglial response, impacting the control of neuroinflammation. These data also suggest that more research is needed to increase our knowledge regarding the potential of these organisms in future neuroprotective therapies.

## 4. Materials and Methods

### 4.1. Sponge Material and Extracts

The marine sponge *A. fulva* (1.5 kg) was collected on 25 October 2021 at Porto da Barra Beach in Salvador, Bahia, Brazil, and packed in a plastic bag containing seawater. The species was identified by Prof. Dr. Emilio Lanna, and a voucher specimen was deposited as UFBAPOR-2479 in the Porifera collection of the Museu de História Natural da Bahia (MHNBA) at the Institute of Biology, Federal University of Bahia, Brazil. Next, the material was immediately moved to the LAPESBI laboratory and washed in running water prior to first being extracted with methanol by maceration (3 × 3 L), followed by ethyl acetate (3 × 2 L). Both organic solutions were combined, and the whole solution was completely evaporated under reduced pressure using a rotary evaporator (50–60 °C) to yield the corresponding crude extract (CE, 3.1 g). In turn, CE was completely suspended in water (500 mL) and extracted with ethyl acetate (3 × 500 mL). The AcOEt phase was then concentrated under reduced pressure (rotary evaporator, 50–60 °C) and immediately dissolved in methanol (500 mL). The methanolic suspension was defatted with hexane (3 × 500 mL), and the remaining methanolic solution was evaporated under reduced pressure (50–60 °C) to yield the methanolic extract (AF-MeOH, 2.5 g).

Part of the AF-MeOH material (2.0 g) was dissolved in MeOH (5 mL) and subjected to an LH-20 Sephadex^®^ column (200 × 5 cm) eluted with MeOH. A total of thirteen fractions (A–M) (10 mL each) were collected. Selected fractions were analyzed by HPLC (Waters Alliance 2690, Milford, MA, USA, XTerra C18 column, 5 μm, 4.6 × 250 mm) under a gradient method (ACN-MeOH-H2O, 5:5:90→50:50:10) with diode array detection (Waters 2996) coupled to a low-resolution quadrupole mass spectrometer with ElectroSpray ionization (Waters ZQ2000, Milford, MA, USA). Thus, all 330 fractions were grouped into thirteen fractions (A–M), taking into account their chromatogram profile similarity, UV absorption regions, and low-resolution masses. Fraction H (1.165 g) was shown to be pure under ^1^H and ^13^C NMR analyses (Agilent Technologies 500/54 Premium Shielded, Santa Clara, CA, USA) and identified as 3,5-dibromoverongiaquinol dimethyl ketal (AF-H1, Figures S1–S7). AF-H1 characterization: white crystals. ^1^H NMR (δ, CD_3_CN, 500 MHz): 6.81 (s, 2H), 6.47 (br s, 1H, NH_α_), 6.00 (br s, 1H, NH_β_), 5.27 (s, 1H, OH), 3.16 (s, 3H, OCH_3_), 3.11 (s, 3H, OCH_3_), 2.49 (s, 2H). ^13^C NMR (δ, CD3CN, 125 MHz): 172.9, 142.6 (2C), 122.7 (2C), 98.0, 71.8, 51.6, 51.5, 45.0. MS/ESI^+^ (*m*/*z*) = 391.7 (6%, M+Na+), 393.7 (12%, M+2+Na+), 395.7 (6%, M+4+Na+), 339.8 (100%). All spectrometric data were in agreement with those previously reported [25,26].

### 4.2. Cell Culture of the PC12 Lineage

The rat pheochromocytoma cell line PC12 was purchased from ATCC (#CRL-1721.1 PC12 ADH, *Rattus norvegicus*, Manassas, VA, USA) and cultured as previously described by Pereira et al. (2017) [27]. Briefly, cells were cultured in DMEM (Cultilab, São Paulo, SP, Brazil), supplemented with L-glutamine (Cultilab, SP, Brazil), 10% inactivated fetal bovine serum (FBS, Cultilab, SP, Brazil), 5% inactivated equine serum (Cultilab, SP, Brazil), 1% penicillin, and 1% streptomycin (Cultilab, São Paulo, Brazil). PC1-12 cells were grown to confluence in 10 mm polystyrene dishes (TPP, Trasadingen, Switzerland), trypsinized (0.05% trypsin and 0.02% EDTA, diluted in phosphate buffer solution (PBS), and replated in a 96-, 24-, or 6-well plate (7.5 × 10^3^ cells/cm^2^) depending on the experiment. All cultures were maintained in an incubator with a humidified atmosphere with 5% CO_2_ and 37 °C.

### 4.3. Microglia Culture

Microglial cells were obtained from the cortex of neonatal Wistar rats (0–2 days old) kept in the vivarium of the Institute of Health Sciences—UFBA, Salvador, Brazil. All experimental procedures were carried out in accordance with the National Institute of Health (GINS) Guide for the Care and Use of Laboratory Animals, approved by the Animal Ethics and Experimentation Committee of the Federal University of Bahia of the Institute of Health Sciences (process number 6731220818). To obtain microglia, primary cultures of glial cells were carried out according to the protocol already well-established in the group [28]. Briefly, after euthanasia, brains from neonatal Wistar rats were mechanically dissociated and resuspended in DMEM supplemented with 10% fetal bovine serum (FBS), 10% equine serum (SE), 4 mM L-glutamine, 100 U/mL of penicillin, and 100 μg/mL of streptomycin. Cells were cultured in flasks coated with poly-D-lysine (25 μg/mL). Upon reaching confluence (7 to 10 days), adherent microglial cells were harvested by shaking at 165 rpm at 37 °C for 3 h. Isolated microglia were seeded in 24- or 6-well plates at a density of 3 × 10^4^ cells/cm^2^ and experiments were performed after 24 h. All cultures were maintained in an incubator with a humidified atmosphere with 5% CO_2_ and 37 °C.

### 4.4. Treatments

AF-MeOH and the compound AF-H1 were diluted according to their mass in dimethylsulfoxide (DMSO; Sigma-Aldrich, St. Louis, MO, USA) to form stock solutions at concentrations of 50 mg/mL and 100 mM, respectively, and kept at −4 °C. The solution for use was obtained at the time of treatment in DMEM without supplementation. Cytotoxicity of AF-MeOH and AF-H1 to PC12 cells was tested at concentrations of 0.1, 1, 10, 100, and 200 μg/mL and 0.1, 1, 10, 50, and 100 μM, respectively. The choice of these concentrations was based on studies conducted with other *Aplysina* sp. [9] and brominated compounds [28] to proliferative cells, and cell viability was determined after 24 h treatments. The protection of PC12 cells by AF-MeOH and AF-H1 to LPS cytotoxicity was tested at concentrations of 0.1–10 μg/mL and 0.1–10 μM, respectively. For this, PC12 cells were treated with LPS (5 μg/mL) for 12 h and treated with or without AF-MeOH (1 and 10 μg/mL) or AF-H1 (1 and 10 μM) for an additional 24 h. The concentrations of AF-MeOH and AF-H1 adopted in this assay were based on the results of the cytotoxicity assays on PC12 cells. Inflammatory damage with LPS has long been adopted for in vitro studies, as previously described [14,15]. This bacterial toxin interacts with Toll-like receptors, leading to the early activation of the NF-κB, IRF3 and MAPK kinase pathways, which in a series of phosphorylation reactions of cellular targets, gives rise to the expression of numerous proinflammatory genes. Control cultures were treated with medium containing DMSO in volumes equivalent to the highest concentration adopted in each assay. 

To characterize the effect of AF-MeOH or AF-H1 treatments on neuronal cells exposed to LPS inflammatory damage on the inflammatory profile of microglia, PC12 cells were cultured in 6-well plates, treated with LPS (5 μg/mL) for 12 h, and then treated or not with AF-MeOH (1 and 10 μg/mL), or AF-H1 (1 and 10 μM), or maintained in control conditions (DMSO 0.1%). After that, the conditioned medium of the PC12 cultures were collected and centrifuged at 2000 rpm for 5 min, transferred to sterile tubes and stored at −80 °C. Microglia growing in 6-well plates were then treated with MC from PC12 cells under control conditions, treated with LPS alone, or treated with both LPS and AF-MeOH or LPS and AF-H1 for 24 h. Three independent experiments were performed under each condition.

### 4.5. Cell Viability by Assays

#### 4.5.1. MTT Test

Cell viability was assessed using the 3-(4,5-dimethylthiazol-2-yl)-2,5-diphenyltetrazolium bromide (MTT; Sigma, St. Louis, MO, USA) test. The experiment was carried out in 96-well plates (Kasvi, Sao Jose dos Pinhais, Brazil) with cultures of cells of the PC-12 lineage and microglia. Cells were incubated with DMSO control and respective treatments for 72 h. The treatment time was chosen based on previous studies by Lima et al., 2017. Cell viability was quantified using the conversion of yellow MTT by live cell dehydrogenase to purple formazan MTT. Control and treated cells were incubated with MTT (1 mg/mL) for 2 h. Subsequently, cells were lysed with 20% (*w*/*v*) sodium dodecyl sulfate (SDS) and 50% (*v*/*v*) dimethylformamide (DMF) (pH 4.7). The plates were incubated overnight at 37 °C to dissolve the formazan crystals. The optical density of each sample was measured at 492 nm using a spectrophotometer (Varioskan TM Thermo Plate-Reader^®^, Thermo Fisher Scientific Inc., Vanta, Finland). In cytotoxicity assays after 72 h of treatment for all extracts, eight replicate wells were performed for each condition. In cytotoxicity assays after 24 h of treatment with AF-H1, three independent experiments were performed with eight replicate wells for each analysis. MTT test results are expressed as percentages of viability of treated groups compared to control groups (considered 100%).

#### 4.5.2. Trypan Blue Cell Viability Test

To analyze the effect of AF-MeOH and AF-H1 on the viability of PC12 cells subjected to inflammatory damage, the blue exclusion test was used, which evaluates the integrity of the cell membrane. After 24 h of treatment, the supernatant was collected and the cells were detached with trypsin, centrifuged at 2000 rpm for 10 min, resuspended in medium and stained with Trypan blue (0.1%), incubated for 10 min, and the number of viable and non-viable cells (blue) calculated in 10 μL samples of cell suspension in a Neubauer chamber using optical microscopy. Cell viability was determined based on the exclusion capacity of the dye, where a blue halo formed around viable cells, while non-viable cells were stained blue. Quantification of the percentage of cells was performed by evaluating the ratio of viable cells to the number of total cells.

#### 4.5.3. Analysis and Cell Viability by Propidium Iodide

The cell viability of microglia subjected to MC treatment of PC12 cells was analyzed using the propidium iodide (PI) test. This technique allows for the analysis of viability through plasma membrane integrity in cell cultures. After the cell cultivation period, the supernatant was removed and then the PI was added at 5 μg/mL diluted in DMEM culture medium and incubated for 1 h in an oven at 37 °C and 5% CO_2_. Afterward, the IP solution was removed, and the wells were washed 3× with PBS-0.6% glucose solution. The cells were analyzed and photographed under a fluorescence microscope (Leica DMIL Led Fluor/Leica DFC7000 T Camera, Mannheim, Germany). Three images of each treatment were taken and evaluated using ImageJ software (Wayne Rasband; National Institutes of Health, Kensington, MD, USA, https://imagej.net/software/imagej/). The results were evaluated through the relationship between the total number of cells and fluorescence intensity.

### 4.6. Cell Morphology Analysis

Morphological changes in the PC12 neuronal cells and microglia were observed and documented after the different treatments by contrast interference microscopy (Nikon^®^ Microscope coupled to the AxioCam HRM camera, Tokyo, Japan). 

To evaluate the microglial activation and inflammatory phenotype, microglia cultures subjected to treatments with CM from PC12 cultures in different conditions were fixed with cold methanol for 20 min at −20 °C. Fixed cells were incubated overnight in a humid chamber with solutions of primary antibodies for Iba-1 (Wako (Louisville, KY, USA)—1:200) and CD-68 (Abcam (Cambridge, UK)—1:200) in PBS containing 1% Triton X100. After three washes with PBS, the cells were incubated with the secondary antibodies Alexa Fluor 488 (Mouse) and Alexa Fluor 594 (Rabbit) (1:1000, Life Technologies, Carlsbad, CA, USA) for 3 h. Subsequently, the nuclear chromatin was labeled with the DNA intercalator 4′6′-diamino-2-phenyl-indole (DAPI-Eugene) at 5 μg/mL for 10 min. Cellular analyses and photomicrographs were performed using a fluorescence microscope (Leica DMIL Led Fluor/Leica DFC7000 T Camera). For each condition, eight photomicrographs were taken, and quantification was analyzed using ImageJ software (Wayne Rasband; National Institutes of Health, USA). 

### 4.7. Statistical Analysis

The data were statistically analyzed using the GraphPad Prism 8.00 software (GraphPad, San Diego, CA, USA) for Windows. Results were expressed as the mean ± SEM. One-way analysis of variance (ANOVA) followed by the Kruskal–Wallis test were used to determine significant differences between groups, differing in only one parameter. *p* values less than 0.05 were considered significant.

## Data Availability

Data are contained within the article.

## Supplementary Materials

The following supporting information can be downloaded at: https://www.mdpi.com/article/10.3390/md22060235/s1, Figure S1: UV (220 nm); Figures S2 and S3: ESI+; Figures S4–S7: ESI−.
