# Supplementary material for: Methanolic Extract and Brominated Compound from the Brazilian Marine Sponge Aplysina fulva Are Neuroprotective and Modulate Inflammatory Profile of Microglia
Source: Mar Drugs. 2024 May 22;22(6):235. doi: 10.3390/md22060235 (PMC11204514; doi:10.3390/md22060235)
Supplement: Supplementary file 1 [file marinedrugs-22-00235-s001.zip › marinedrugs-2973252-supplementary.pdf]

Grouped fractions H

Figure S1

UV (220 nm)

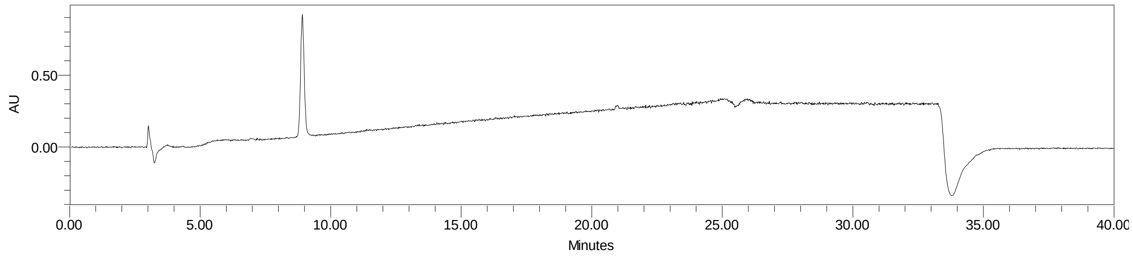

Figure S2

ESI+

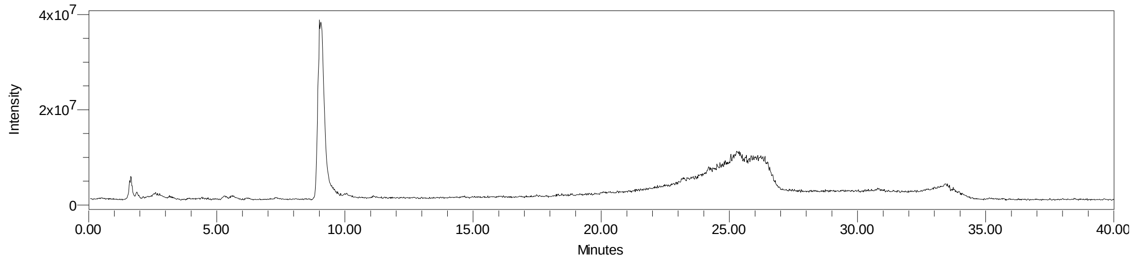

Figure S3

t_R 9,025 minutos

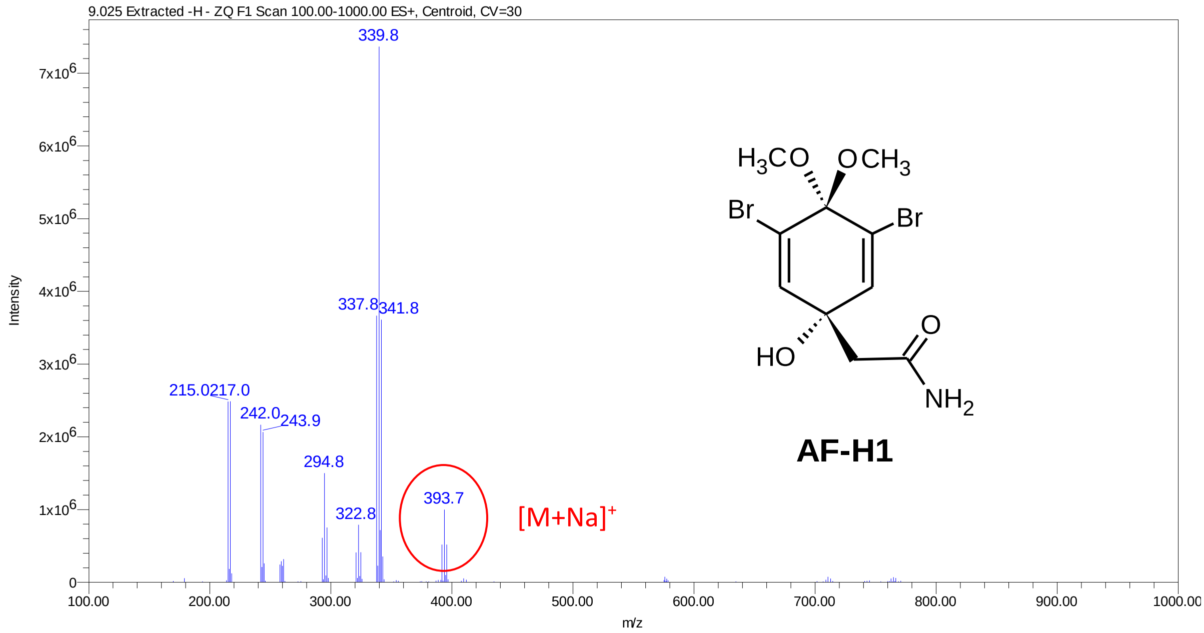

Figure S4

ESI-

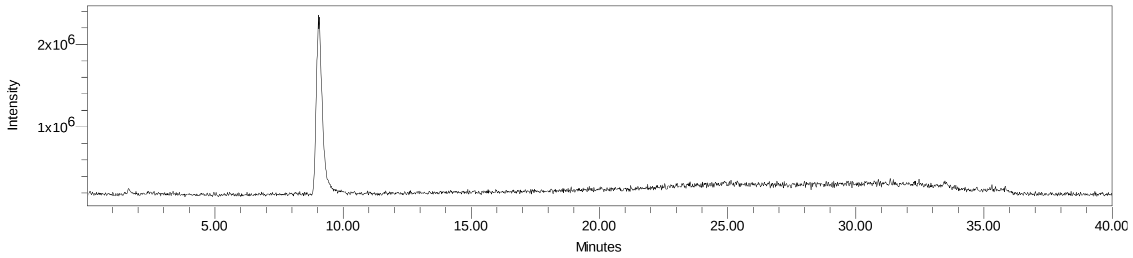

Figure S5

t_R 9,055 minutos

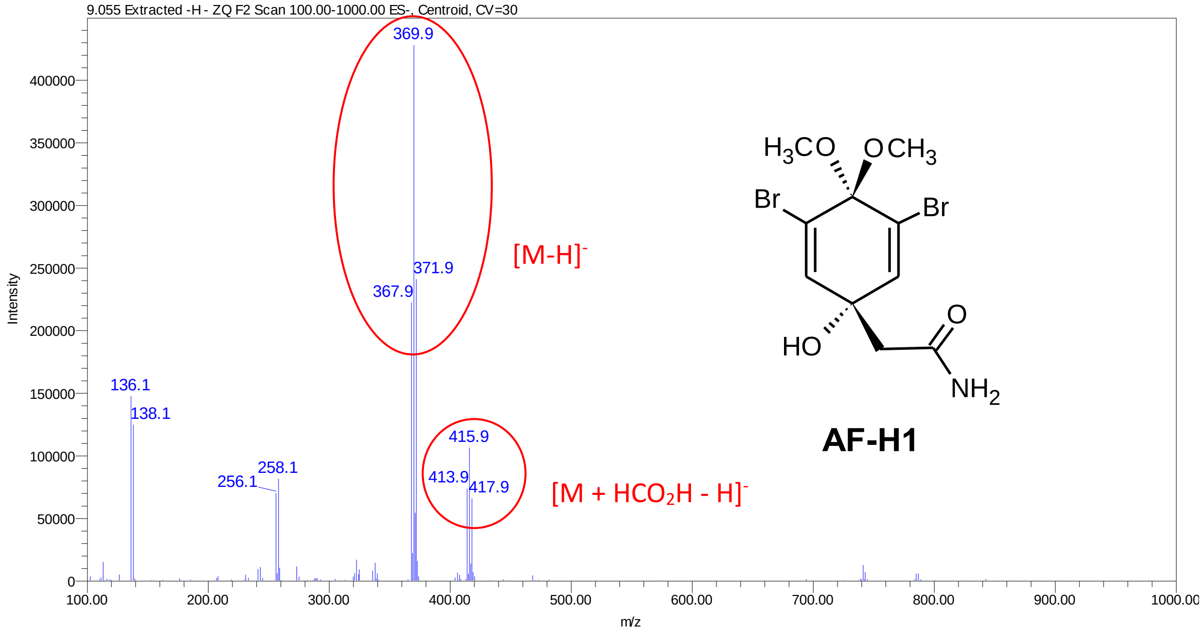

Figure S6

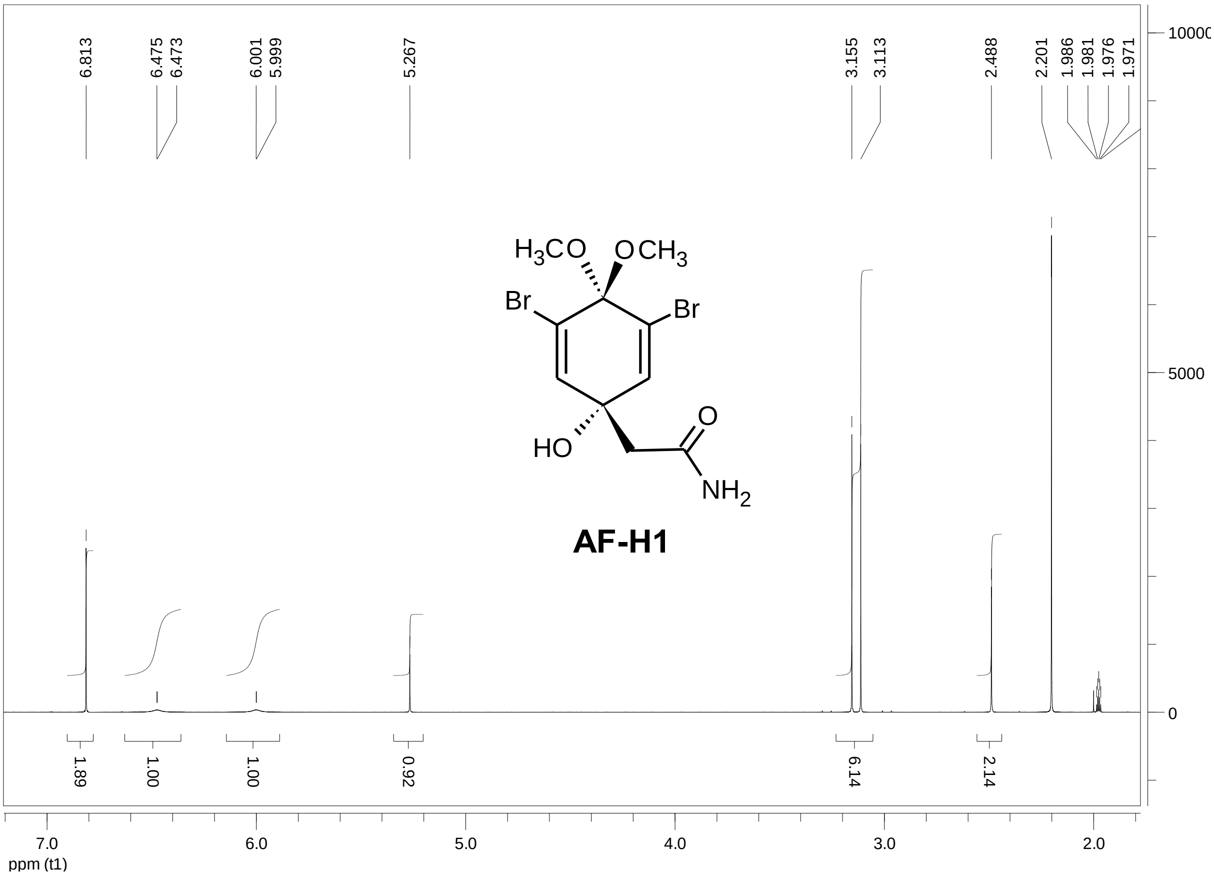

^{1}H NMR spectrum of **AF-H1** (500 MHz, CD_3CN, ppm)

Figure S7

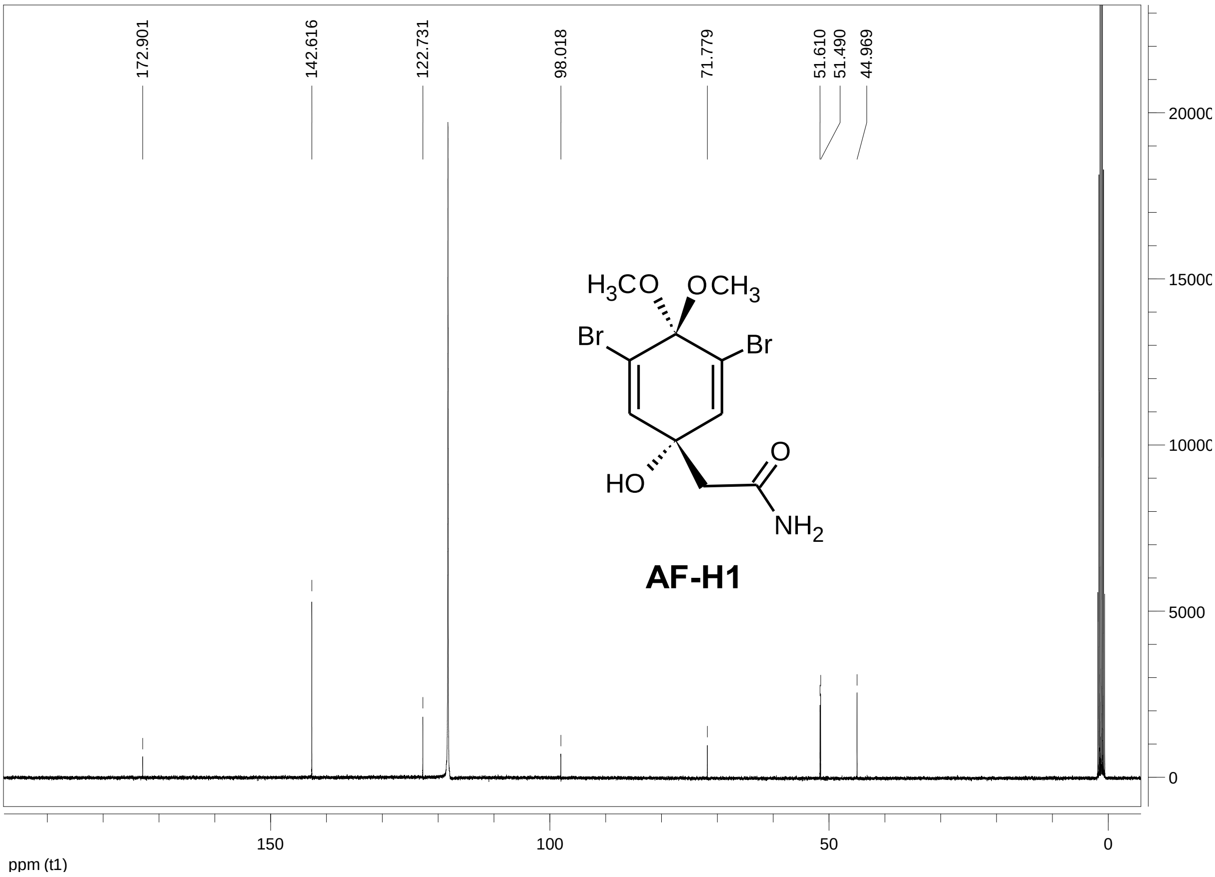

^{13}C NMR spectrum of **AF-H1** (125 MHz, CD_3CN, ppm)
